# Associations between trunk mobility, pain, and quality of life in individuals with chronic low back pain treated with different therapeutic protocols: Potential clinical parameters

**DOI:** 10.1177/10538127251358730

**Published:** 2025-07-14

**Authors:** Robbert Van Amstel, Karl Noten, Shaun Malone, Peter Vaes

**Affiliations:** 1 Department of Human Movement Sciences, Faculty of Behavioural and Movement Sciences, Amsterdam Movement Sciences, Vrije Universiteit Amsterdam, Amsterdam, The Netherlands; 2 Fysio Science Department, Fysio Physics Group, IJsselstein, The Netherlands; 3 Department of Rehabilitation Sciences and Physiotherapy (MOVANT), University of Antwerp, Antwerpen, Belgium; 4 Faculty of Physical Education and Physiotherapy, Vrije Universiteit Brussel, Brussels, Belgium

**Keywords:** Low back pain, quality of life, range of motion, articular, correlation of data

## Abstract

**Background:**

Physiotherapy guidelines for managing low back pain (LBP) recommend the use of validated measures such as trunk mobility, pain intensity, and questionnaires to evaluate function. However, the relationship between these clinical parameters remains insufficiently understood.

**Objective:**

To investigate associations between trunk mobility, mobility-dependent pain, and quality of life (QOL) as potential clinical parameters in individuals with nonspecific chronic low back pain (NSCLBP).

**Methods:**

A secondary analysis was conducted on data from 51 individuals with NSCLBP enrolled in a randomized trial comparing the 4xT method and physiotherapeutic-guided exercise. Both groups completed a six-week rehabilitation program with two sessions per week, followed by a six-week therapy-free period. Trunk range of motion, mobility-dependent pain, and perceived health were analyzed as predictors of QOL using correlation and linear regression.

**Results:**

Increased trunk mobility and elevated perceived health are positively associated with QOL in individuals with NSCLBP. Higher levels of mobility-dependent pain are negatively associated with QOL. The interaction between trunk mobility and changes in mobility-dependent pain intensity did not have an additional impact on QOL. Overall, our findings indicate that these associations were moderate or occasionally weak.

**Conclusions:**

Trunk mobility, mobility-dependent pain, and perceived health are relevant clinical predictors of QOL in individuals with NSCLBP. These findings highlight the importance of assessing both objective physical function and subjective pain perception when evaluating rehabilitation outcomes. Targeting trunk mobility and mobility-dependent pain in LBP treatment may lead to more personalized care and improved QOL. Including these measures should be standard practice when assessing rehabilitation effectiveness.

**Clinical Trial Registration Number:** NCT03309540

## Introduction

Low Back Pain (LBP) has emerged as the leading contributor to years lived with disability globally.^1,2^ LBP has a prevalence of 1.4% to 20.0%^3^ with a lifetime prevalence of 84%^4^ and is expected to increase due to aging.^5^ It is estimated that approximately 90% of individuals with LBP have an unknown underlying cause,^6,7^ which is highest at the age of 25–65 years.^8^

Researchers commonly conclude that in individuals with LBP, trunk motion patterns play a crucial role in the development and recovery of nonspecific LBP.^9^ Trunk motions in the sagittal plane are widely investigated in LBP and healthy individuals.^10–13^ By finding irregularities in trunk motions, limitations in daily activities can be identified.^14^ The assessment of trunk motions involves measuring the range of motion (ROM), regional movement timing, muscle activation, and the flexion-relaxation phenomenon to understand the association between trunk motion and factors influencing it.^14,15^ Understanding the relationship between trunk motion and bending tasks is crucial for evaluating and diagnosing disorders in lumbopelvic rhythm and functional instability.^16,17^

Individuals with nonspecific LBP often exhibit characteristics such as significantly slower movements, increased back muscle activity, reduced thoracolumbar fascia sliding mobility, and reduced trunk ROM.^18–20^ In addition, research has demonstrated that functional disability, assessed through questionnaires, in individuals with LBP has an impact on both the experienced pain intensity and quality of life (QOL).^21–23^ Hence, clinical guidelines for physiotherapists emphasize the importance of assessing function using validated tools, combining objective measures like trunk motion and ROM with subjective assessments such as pain intensity and patient-reported questionnaires.^24,25^ This recommendation is rooted in the belief that a thorough evaluation of these factors is crucial for determining the most suitable physiotherapeutic interventions. The primary goal is to improve functional disability, health, and enhance overall QOL for individuals dealing with LBP.^24,25^ By considering and addressing trunk motion, ROM, and pain intensity, healthcare providers can tailor their interventions to better meet the specific needs of each LBP patient. This individualized approach aims to reduce functional disability and enhance overall health and QOL, leveraging commonly used parameters in clinical practice.^26,27^ However, most studies that investigated trunk mobility using ROM did not provide information on motion patterns or ROM disturbances, the experienced pain intensity, functional disability, and QOL in individuals with nonspecific LBP,^28^ therefor, there is a lack of research examining the relationship between them.

This study aims to explore the relationship between changes in mobility-dependent pain intensity, trunk ROM, health, and their impact on QOL in 51 individuals with nonspecific LBP. Primarily, the aim of this study was to examine how changes in QOL relate to changes in trunk ROM, mobility-dependent pain intensity, and perceived health. Secondarily, the aim was to consider how the interaction between trunk mobility and mobility-dependent pain intensity might influence these relationships.

## Methods

### Design

This paper presents a secondary analysis of prospectively collected data from a previously registered clinical trial (NCT03309540). This study was a secondary analysis of two therapeutic protocols, based on data obtained in a clinical trial that compared the 4xT method with physiotherapist-guided exercise therapy in individuals with nonspecific chronic LBP (NSCLBP).^29^ The 4xT group received a standardized fascia-focused intervention protocol used by clinicians, consisting of four components: Test (functional diagnostic test), Trigger (fascia tissue manipulations), Tape (elastic taping), and Train (exercise).^30,31^ The test included the Dynamic ArthroMyofascial Translation Test (DAMT-Test).^31^ The DAMT-Test includes a baseline assessment (to distinguish the most painful direction from the less painful direction) and two post-assessments, which involve active trunk motion (most painful direction performed by the patient) combined with ongoing skin displacement (e.g., mediolateral skin displacement to the left vs. right at hight L3, performed by the therapist). Based on the positive effects on trunk ROM and pain levels, the most effective direction of the fascia tissue manipulations (triggers) and elastic tape at the tested location was determined. When the participant could move with an acceptable pain level (pain NRS < 2), the exercises were performed in the less painful direction as determined during the baseline assessmen. The exercise therapy group received physiotherapeutic-guided exercise therapy exercise therapy, which included a structured program. The exercise program focused on exercises aimed at improving joint mobility, promoting muscle growth and strength,^32^ and stabilizing the core, particularly the lumbar multifidus and transverse abdominal muscles, which are essential for trunk stability.^33^ The exercise programs consisted of a combination of machine-based and Pilates exercise. This study spanned 12 weeks (6 weeks on and 6 weeks off physiotherapy), with measurements taken at four evaluation time points: week 0, 3, 6, and 12 (resp. T0, T1, T2, T3) on non-treatment days. Repeated measurements over time facilitated the examination of changes and trends within participants, reflecting the characteristic design of a longitudinal study. The study was conducted in accordance with the Declaration of Helsinki,^34^ and was approved by the Committee of Medical Ethics, B.UN 143201627110. All individuals were informed, had read, and had signed the informed consent.

### Recruitment and participants

The NSCLBP individuals were recruited between October 2017 and December 2019 using advertisements and via direct access consult physiotherapy. Subjects meeting NSCLBP criteria (LBP ≥12 weeks), Pain Numerical Rating Scale (pain NRS) ≥ 4 in either end flexion or extension motion, and aged 25–65 were included. This age was chosen due to the highest LBP disability rates.^8^ Participants were excluded if they presented with any of the following: radiating pain, neurological symptoms (sensory and/or motor), malaise, malignancy, corticosteroid use, osteoporosis, spondylitis, stenosis, arthritis, vertebral fracture, and severe deformity.^24^ Fifty-one NSCLBP individuals enrolled; randomization distributed them between the exercise group (n = 24) and the 4xT group (n = 27). The allocation was performed at random using a computer-generated randomized table. Importantly, all collected data, irrespective of group allocation, were utilized in subsequent relationship analysis.

### Sample size

A power analysis using MATLAB (version 2023b, The MathWorks, Inc., Natick, MA, USA) was specifically conducted to determine the sample size requirements for correlation analysis, which was crucial for assessing the relationships between variables in this study.^35^ The analysis indicated that to achieve adequate statistical power (1-β = 0.80, α = 0.05, expected effect size = 0.23), a minimum of 144 data points was necessary to reliably detect significant correlations.

### Outcomes

The QOL, trunk ROM, and pain intensity were measured and documented during the evaluation on a non-treatment day. Trunk mobility and pain intensity were measured simultaneously using a standardized functional test procedure, which was referred to as the mobility-dependent pain assessment.^29,36^ To minimize bias and ensure objectivity in data collection, the trunk ROM data was evaluated by an independent assessor who remained blinded to the entire study and the operator. The trunk ROM, pain intensity, and QOL data were recorded separately and concealed by the research assistant.

#### Quality of life and health status

In this research, the participants’ QOL was assessed using the EQ-5D-3L index and the VAS for health status (H-VAS). The use of this tool was pre-registered (Euroqol Group, reg. nr. EQ: ID16666). Each aspect of QOL, including mobility, self-care, daily activities, pain/discomfort, and anxiety/depression, was assessed through individual questions within the EQ-5D-3L index. These questions consisted of three levels of severity: no problems, some or moderate problems, and extreme problems/unable to perform. Each level received a distinct score, which could be aggregated to provide an overall assessment of health status, often expressed as weight. This assessment of QOL was derived using a specific formula. These scores were based on evaluations from the general population, considering all potential combinations of QOL across various dimensions (euroqol.org). Higher index scores indicated better QOL. A score of 1 represented perfect QOL, while scores below 1 indicated some level of quality impairment. Higher scores on the H-VAS represented better self-perceived health. The EQ-5D-3L and H-VAS were both reported to be valid and responsive scales, exhibiting moderate to good correlations (0.39 to 0.59) with each other.^37^

#### Trunk range of motion and mobility-dependent pain intensity

A standardized functional test was used to assess trunk mobility and pain intensity to ensure uniform execution.^36,38^ The sagittal trunk ROM in degrees was evaluated using a single baseline bubble inclinometer (Model 10602, Fabrication Enterprise Inc. USA). A reference tape (Leukoplast Classic, BSN Medical, Hamburg, Germany) between two spinal processes was used, and trunk ROM was measured with the tape between the inclinometer's arc. Trunk ROM (mean of 3 measurements) was quantified by trunk flexion ROM (FROM) and trunk extension ROM (EROM). For trunk ROM assessments, the inclinometer was placed on L1T12 to measure total mobility. For isolated FROM (isFROM), the inclinometer was placed on S1S2, and the difference was calculated (isFROM = L1T12-S1S2) to measure lumbar mobility. The inclinometer was set to 0° with the individual standing upright. Reliability of trunk ROM was ensured by assessing interrater agreement between the operator and a blinded assessor during all measurement sessions [(FROM, ICC = .98, CI95% .95–.99) & (EROM, ICC = .97, CI95% .93–.99)].^29^

The intensity of pain experienced during both FROM and EROM was recorded as mobility-dependent pain, and documentation occurred after the completion of the respective movements. To quantify the intensity of pain, the NRS was utilized, which rated pain from 0 (no pain) to 10 (most experienced pain).^39^ The pain NRS showed high reliability in assessing LBP and had minimal bias (mean difference = 0.33).^40^

#### Data transformation

Since we were interested in whether the change in QOL was related to the eventual change in trunk ROM, mobility-dependent pain, and health status, we needed to transform the data by calculating the difference between the follow-up measurements at T_x_ (T1, T2, T3) and the baseline data at T0 $\left(T_{x}-T0\;\right)$ for each variable. Because the pain NRS was assessed in relation to trunk end ROM, our analysis included an interaction term between these variables. This interaction term was used as a control variable to examine its effect on changes in QOL. Specifically, the interaction term was calculated as follows:

Flexioninteractionterm=FROM×FlexionPNRS

Extensioninteractionterm=EROM×ExtensionPNRS

IsolatedFlexioninteractionterm=isFROM×FlexionPNRS

Since our dependent variable, QOL, was a continuous measure, we treated the interaction term between ROM (which was continuous) and pain NRS (which was ordinal) as continuous in our linear regression analysis.^41^ Multicollinearity was not detected, either among the interaction terms or between the interaction terms and ROM or PNRS (r < .07), indicating that the assumptions for linear regression were met and no further transformation was required.^42^

### Statistical analysis

Descriptive statistics, including means and SD, were obtained. Given the non-normal distribution of our dependent variable, EQ-5D-3L, Spearman's rank correlation coefficient (rs) was selected for assessing the association between the different outcome measures. The independent variables included FROM, EROM, isFROM, flexion and extension mobility-dependent pain NRS, and HVAS.

A multiple linear regression analysis was employed to explore how much variation in EQ-5D-3L was determined by the trunk ROM and mobility-dependent pain NRS when controlling for specified interaction terms (flexion or extension). Specifically, we performed multiple regression analysis using two enter models, one for extension and one for flexion, each with one dependent variable (QOL) and three independent variables (ROM, PNRS, and ROM × PNRS) entered simultaneously. The linearity of the residuals was confirmed using Q-Q plots, while homoscedasticity was assessed through residual scatterplots. In addition, the residuals for all models (σ < 0.5) fell within the limits of three sigma, supporting the assumptions of normality and linearity for the relationships between the dependent variable and the covariates.^43^ These methods ensured that the assumptions of linearity and equal variance of residuals were met.

The following correlation classifications were used: no relation (0), weak relation (0.1 to 0.3), moderate relation (0.4 to 0.6), strong relation (0.7 to 0.9), and perfect relation (1). This interpretation of rs values was widely utilized in scientific contexts, particularly in biopsychosocial research.^44^

## Results

In total, 153 data points for each variable were included, and no missing data were present. The mean baseline characteristics were presented in Table 1.

**Table 1.** Baseline characteristics.

	N (cases)	Mean	±(SD)
isFROM	153	36.93	12.23
FROM	153	91.04	19.41
FPNRS (0–10)	153	4.29	2.17
EROM	153	18.53	8.24
EPNRS (0–10)	153	4.34	2.50
H-VAS (0–100)	153	55.12	18.01
EQ-5D-3L	153	.548	.279
Male/Female	29/22	-	-
Age	51	45	10

Abbreviations: is, isolated; FROM, Flexion Range of Motion; EROM, Extension Range of Motion; FPNRS, Flexion movement-dependent Pain Numerical Rating Scale; EPNRS, Extension movement-dependent Pain Numerical Rating Scale; H-VAS, perceived health Visual Analog Scale; EQ-5D-3L, quality of life EuroQol-5 Dimensions-3 Levels.

**Figure 1.**
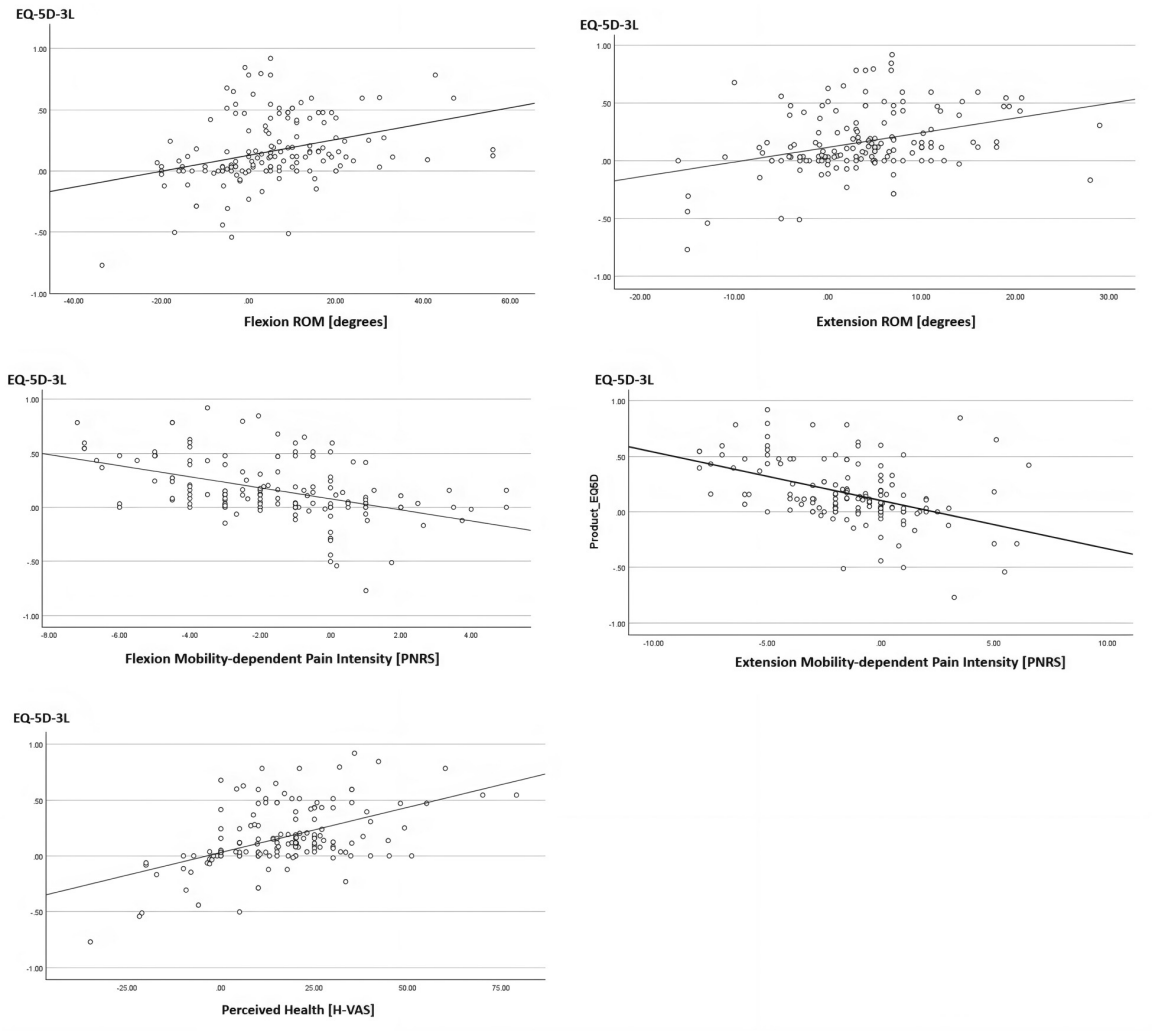
Quality of life vs. trunk mobility, mobility-dependent pain, and perceived health. Abbreviations: ROM, Range of Motion; PNRS, Pain Numerical Rating Scale; H-VAS, perceived health Visual Analog Scale; EQ-5D-3L, quality of life EuroQol-5 Dimensions-3 Levels.

We observed a moderate positive relationship between FROM and EQ-5D-3L scores (rs = .41, p < .001). This suggested that an increase in flexion mobility was related to an improvement in the QOL for individuals with NSCLBP. In addition, a weak positive correlation was observed between EROM and EQ-5D-3L scores (rs = 0.34, p < .001), as well as between isFROM and EQ-5D-3L scores (rs = .27, p < .001). A moderate negative correlation was found between mobility-dependent flexion pain NRS and EQ-5D-3L scores (rs = −.48, p < .001). This suggested that a reduction in pain at the end ROM corresponded to better QOL. Next to this, a moderate negative correlation was found between mobility-dependent extension pain NRS and EQ-5D-3L scores (rs = −.45, p < .001), revealing that a reduction in pain experienced at the end of trunk extension corresponded to an overall better QOL for individuals with NSCLBP. In addition, a moderate positive relationship was identified between HVAS and EQ-5D-3L scores (rs = .47, p < .001) (Figure 1).

**Figure 2.**
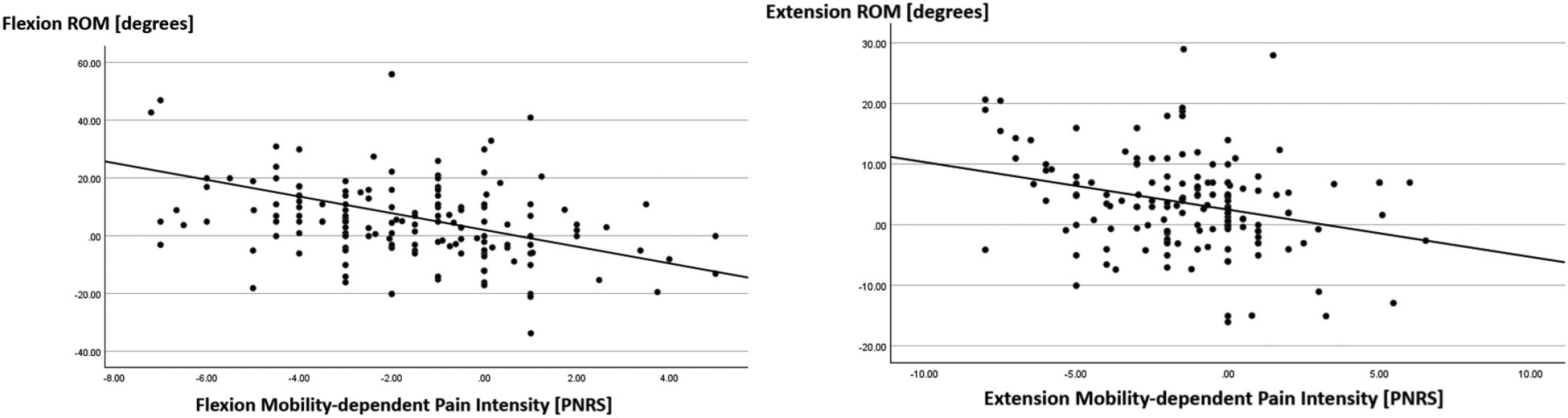
Trunk mobility vs. mobility-dependent Pain. Abbreviations: ROM, Range of Motion; PNRS, Pain Numerical Rating Scale.

In the quest to examine whether changes in trunk mobility were linked to changes in pain intensity, a moderate negative relationship was observed between FROM and mobility-dependent flexion pain NRS (rs = −.40, p < .001). In addition, a weak positive relationship was observed between EROM and mobility-dependent extension pain NRS (rs = .24, p < .003) (Figure 2). This suggested that an increase in trunk mobility was related to a reduction in pain experienced at the end of trunk motion.

The multiple linear regression model, including variables FROM, mobility-dependent flexion pain NRS, and the flexion interaction term, explained 24.6% of EQ-5D-3L variability (R² = .246, p < .001). While no significant association was found for the flexion interaction term, both changes in FROM (β = .230, p < .001) and mobility-dependent flexion pain NRS (β = −.394, p < .001) were significantly associated with EQ-5D-3L. Similarly, the model with variables EROM, mobility-dependent extension pain NRS, and the extension interaction term significantly explained 26.7% of EQ-5D-3L variability (R² = .267, p < .001). The change in EQ-5D-3L was not associated with the extension interaction term, whereas both EROM (β = .297, p < .001) and mobility-dependent extension pain NRS (β = −.440, p < .001) demonstrated significant associations. In addition, similar findings were observed for the model including variables isFROM, mobility-dependent flexion pain NRS, and the isolated flexion interaction term (R² = .235, p < .001). No significant association was found for the isolated flexion interaction term, while isFROM (β = .204, p < .001) and mobility-dependent flexion pain NRS (β = −.409, p < .001) exhibited a significant association with EQ-5D-3L. This meant that changes in ROM and changes in mobility-dependent pain intensity both significantly affected QOL, but together they did not have an additional impact on it.

## Discussion

In the management of LBP, clinical guidelines for physiotherapists state the significance of evaluating trunk mobility, pain intensity, functional disability, and QOL.^24,25^ The discrepancy between the mean mobility-dependent pain NRS scores (flexion pain NRS:4.29 ± SD 2.17, extension pain NRS: 4.34 ± SD 2.50) and the inclusion criterion of ≥ 4 can be partly explained by our requirement for a pain NRS of ≥ 4 in either flexion or extension. This variability within individuals across different movement directions may contribute to the observed SD in our baseline statistics, emphasizing the need to consider individual responses to specific movements when assessing pain intensity.^30,45,46^ The study results revealed that reduced mobility-dependent pain, increased trunk mobility, and better perceived health were all associated with improvements in the QOL for those with NSCLBP. Moreover, increased trunk mobility is associated with mobility-dependent pain in individuals with NSCLBP. In general, our findings show associations that are moderate or sometimes weak.

The study results indicate that increased flexion mobility (rs = .41) and extension mobility (rs = .34) are positively associated with QOL in NSCLBP individuals (moderate association). Moreover, it indicates that a reduction in mobility-dependent pain, measured for both end trunk flexion (rs = −.48) and end trunk extension (rs = −.45), is negatively associated with QOL (moderate association) in individuals with NSCLBP. Research has demonstrated that functional disability in individuals with LBP has an impact on both the experienced pain intensity and QOL.^21–23^ However, these studies assessed the functional disability and pain intensity through questionnaires.

In this study, we used a standardized functional test method in assessing trunk flexion mobility as well as mobility-dependent pain.^36^ Assessing pain intensity at the end of trunk motion provides insights into how pain can affect an individual's trunk mobility. This evaluation method is thought to help in understanding the impact of pain on functional mobility.^12,29,31^ The literature consistently highlights a mutual relationship between trunk mobility and pain intensity.^47,48^ The trunk mobility in this study was measured by placing the inclinometer at the height of L1T12, capturing the total trunk ROM, including hip mobility in this measurement method.^49^ Despite measuring high intertester reliability and literature reporting strong reliability and validity with small measurement errors, this method of measuring trunk mobility has its limitations.^49,50^ Hence, we standardized the measurement method to increase the reliability and consistency of our findings, ensuring an accurate assessment of trunk mobility across all individuals.^36^ In individuals with LBP, hip mobility disorders are often associated with higher pain intensity. This may explain why we did not find a significant association between isFROM and flexion mobility-dependent pain, as isFROM measures the lumbar spine angle. However, our study results suggest a weak to moderate association between an increase in trunk extension mobility (r_s_ = −.24) and flexion mobility (r_s_ = −.40) with favorable changes in mobility-dependent pain intensity (mobility increases when pain decreases). Similarly, in a laser therapy study, pain intensity measured with VAS showed varied and significant negative correlations for lumbar FROM (r = −.43), though not for EROM.^51^ Our findings were based on assessing mobility-dependent pain at four time points (week 0, 3, 6, and 12) in individuals with NSCLBP^29^; in contrast, the referenced study employed a questionnaire to measure pain intensity before and after 18 therapy sessions.^51^ The assessment of pain intensity (sitting vs. standing) was not reported, which could potentially influence the relationship between pain intensity and trunk ROM.^51^

The moderate correlations with the change in QOL reveal that it is not solely caused by changes in perceived health, trunk ROM, and mobility-dependent pain. Subsequently, it was confirmed in regression models that the interaction terms were non-significant, affirming that QOL is affected by the individual contributions of changes in trunk ROM and mobility-dependent pain, influenced by other factors. Among others, alterations in thoracolumbar fascia deformability and/or back muscle stiffness could potentially contribute to changes in pain intensity and trunk mobility. A flexion relaxation study suggests that increased lumbar muscle activity, particularly in the subgroup characterized by ‘guarded’ movement, is associated with higher levels of pain intensity.^52^ In addition, ultrasound research has revealed a substantial reduction in thoracolumbar fascia sliding mobility during trunk motion in individuals with LBP compared to that in healthy controls.^18,19^ The sliding mobility magnitude of the thoracolumbar fascia over the paraspinal muscles is correlated with the thoracolumbar fascia thickness (r = −.45), its echogenicity (r = .28), FROM (r = .36), and EROM (r = .41),^18^ and paraspinal muscle activity deviations.^53^ The relationship between biomechanical factors and QOL in individuals with NSCLBP is not fully understood. The relationship between biomechanical factors and QOL in individuals with NSCLBP is not fully understood. However, age may act as a mediating factor due to its association with structural changes in lumbar anatomical morphology and function.^54,55^ These age-related changes could potentially influence the relationship between biomechanical factors and QOL, highlighting the need for further research to clarify this interaction. While we did not include age as a covariate due to the risk of overfitting with our limited sample size, we acknowledge that age may act as a potential confounding factor.^56^

In summary, our results reveal that alterations in biomechanical factors are associated with improvements in QOL, serving as a determinant for psychosocial factors.^57^ As others have stated, it might be essential not to overlook the biomechanical aspects within the biopsychosocial context.^58–60^ Consequently, an urgent demand exists for comprehensive investigations to test the relationship between biomechanical factors and the alterations in QOL experienced by this population.

### Limitations

While providing insights into the relationship between mobility-dependent pain intensity, trunk mobility, health status, and QOL in NSCLBP individuals, this study has limitations. The absence of a functional disability questionnaire neglects crucial information about daily activities, essential for changes in QOL. As a secondary analysis dependent on data quality from an interventional study, potential inconsistencies may affect precision. The fixed sample size, not originally intended for a correlation study, could impact statistical power and generalizability; nevertheless, our sample size (153) surpasses the minimum requirement of 144 data points for this analysis.

## Conclusion

This study emphasizes the importance of evaluating mobility-dependent pain, trunk mobility, and overall health to comprehend their relationships with LBP-related QOL. This insight is essential for the development of individualized rehabilitation strategies. Notably, improvements in perceived health, increased trunk mobility, and the alleviation of mobility-dependent pain intensity correlate directly, albeit weakly to moderately, with an enhanced QOL. These findings imply that interventions aimed at enhancing these factors can positively influence individuals experiencing LBP. However, further research is essential to gain a more nuanced understanding of the intricate interactions among these factors and others.
